# Enhancing immigrant families’ mental health through the promotion of structural and community-based support

**DOI:** 10.3389/fpubh.2024.1382600

**Published:** 2024-05-01

**Authors:** Bonnie D. Kerker, R. Gabriela Barajas-Gonzalez, Natalia M. Rojas, Jennifer M. Norton, Laurie M. Brotman

**Affiliations:** ^1^Department of Population Health, New York University Grossman School of Medicine, New York, NY, United States; ^2^Department of Child and Adolescent Psychiatry, New York University Grossman School of Medicine, New York, NY, United States

**Keywords:** mental health, immigrant health, structural intervention, prevention, health promotion, health equity, maternal support, family-focused policy

## Abstract

Immigrant communities in the United States are diverse and have many assets. Yet, they often experience stressors that can undermine the mental health of residents. To fully promote mental health and well-being among immigrant communities, it is important to emphasize population-level policies and practices that may serve to mitigate stress and prevent mental health disorders. In this paper, we describe the stressors and stress experienced by immigrant families, using Sunset Park, Brooklyn as an example. We discuss ways to build structures and policies in support of equitable environments that promote mental health at the population level and enable families and their children to thrive.

## Introduction

Immigrants to the United States (U.S.) and their U.S.-born children numbered approximately 87.7 million people, or close to 27 percent of the population, in 2022, an increase of approximately 14.7 million (or 20 percent) from 2010 (1). In the United States, immigrant communities are diverse, have many assets and often benefit from personal resilience, strong family connectedness, faith-based supports, and cultural pride (2). But, many immigrant communities face numerous challenges, due in large part to environmental and structural inequities which are often experienced as stressors that can undermine the mental health of residents (3). For example, immigrants in the U.S. have often experienced trauma from conditions in their home countries, discrimination in the U.S., and struggles with U.S. immigration processes and policies (4). Further, many immigrant communities experience economic hardships (5), and those who do not have authorized legal status (e.g., those who are undocumented, those who are applying for asylum) are often left feeling uncertain about what benefits they are eligible for and how receiving benefits might impact them in the future (6). When immigrant parents experience compromised mental health, their children can have long-term negative consequences as well (7).

The COVID-19 pandemic exacerbated stress and mental health inequities nation-wide (8, 9), catalyzing the need to re-examine public investments in mental health promotion, prevention and treatment, especially in families from historically marginalized populations. Scholars and advocates are increasingly recognizing the potential of population-level policies and programs to prevent mental health disorders and reduce inequities (10). In this manuscript, we argue that inclusive policies and tailored programming for families are critical to addressing inequities, decreasing stress, and preventing the escalation of mental health problems in immigrant communities. We describe a range of policies and programs that could help promote mental health at the population-level and lead to a more equitable society.

The lived experiences of immigrant groups in the United States vary widely given differences in country of origin, eligibility for discretionary legal status or citizenship, and time since immigration, as well as the immigration-related policies and practices in the city and states in which they live. This manuscript considers the extant literature on stress and mental health in diverse immigrant communities throughout the United States. At the same time, it highlights the experiences of Chinese and Latinx immigrant families in one community in New York City (NYC) as a way to consider strategies for applying inclusive policies and tailoring programming based on immigrant group characteristics and local context. NYC is one of more than 180 cities and counties in the U.S. that is a sanctuary city (11) (with limits on whether and how officials share information with the Federal government about non-citizens) (12) and New York State has relatively inclusive policies impacting immigrant families, especially compared to states such as Arizona and Texas (13). We focus on the neighborhood of Sunset Park, Brooklyn in NYC because the authors work with and in this community on a place-based initiative with a strong mental health focus called Together Growing Strong (TGS). TGS aims to strengthen the health, well-being, and development of children in Sunset Park by supporting families, educators, health practitioners, and community stakeholders. Two authors (BK and LB) are on the TGS leadership team and three oversee distinct aspects of the work (NR, RB-G, and JN). Our perspective in this manuscript is informed by our work in this community.

## Stress and mental health in immigrant communities

Immigrants make up 14% of the U.S. population. In 2022, nearly one-third of immigrants had entered the U.S. since 2010. More than one-quarter (27%) of immigrants were reported to be Asian, 20% White, and 9% Black; 44% were of Hispanic or Latinx origin. About one-quarter of immigrants living in the U.S. in 2022 were born in Mexico and 6% were born in China (1). The vast majority of immigrants in the U.S. (83%) speak a language other than English at home, with Spanish being most common (14). A large majority (96%) of the immigrant civilian labor force is employed, yet immigrants have slightly higher rates of poverty than those born in the U.S., and one-quarter of immigrants have less than a high school diploma (1).

Similar to many immigrant communities throughout the country, Sunset Park includes vibrant enclaves of immigrants from China and Latin America, with inter-generational families and communities that are tight knit. More than 26,000 families and 32,000 children under the age of 18 live in Sunset Park. In contrast to the U.S. overall, nearly one-half of Sunset Park’s 130,000 residents were born outside the U.S.; about three out of four residents speak a language other than English at home. The vast majority (93%) of the civilian labor force is employed, but one in five residents in Sunset Park live below the poverty line, more than one-third have less than a high school degree, and 7% live in overcrowded housing (15).

When the COVID-19 pandemic hit, families in many immigrant communities throughout the U.S., including Sunset Park, experienced additional stressors including isolation during quarantine, fear of returning to work as “essential workers” in the healthcare and food industries, grief over illness and death of friends and family, job loss leading to worries about financially supporting a family, enhanced food insecurity, and general uncertainty about school and childcare closures (16). In addition, exclusionary and xenophobic policies, as well as rhetoric blaming China for COVID-19, contributed to an increase in hostility toward immigrants, including bullying, language-based discrimination, and anti-Asian hate, which were linked to worse mental health in immigrant communities (17, 18). There is strong evidence that stress resulting from a broad range of circumstances, including financial hardship, is associated with mental health disorders (19–21). Further, unique stressors faced by immigrants, such as restrictive immigration policies and policing, have been shown to be associated with anxiety and depression (4, 17), contributing to substantial mental health inequities. In fact, in states with more restrictive immigration policies, Latinx immigrants report poorer mental health (13, 22).

In Sunset Park, a 2021 TGS study surveyed pregnant and postpartum women about stressors, perceived stress and mental health, and provides an example of the association between the stress felt by an immigrant community in the wake of the pandemic and mental health symptoms. The study used a convenience sample and recruited women through health centers, social service providers, and texting platforms; 671 women completed surveys online or by telephone in Chinese, Spanish and English languages. About one-third of women surveyed had moderate to severe anxiety scores and one-quarter had moderate to severe depression scores, based on PROMIS scales (23); self-reported worry about basic needs, such as rent and food, was associated with higher depression and anxiety scores.

## Policies to support families and promote equity

Policies that strengthen families’ assets and provide additional support by addressing social determinants of health can be effective in helping caregivers and others avoid and manage stressors that interfere with mental health (24). Studies across states (i.e., Pennsylvania, Arizona, and California) find that social support is associated with decreased mental health symptoms and moderates the association between stress and poor mental health (25–27). In Sunset Park, the 2021 TGS survey found that women who reported “usually” or “always” getting the support they needed when they felt stressed had lower mean depression and anxiety scores than other women.

In this paper, we focus on policies that address financial hardship, as immigrant communities often have high rates of poverty (5) due to underemployment, labor exploitation, and barriers to employment and economic opportunity (28). Paid family leave is one example of a scalable federal policy that supports families and is associated with decreased postpartum psychological distress symptoms among mothers with infants (29). Yet, the U.S. is the only developed country that does not guarantee a period of paid and job-protected leave for new mothers (29), thus bypassing an opportunity to promote equity. Further, unconditional cash transfers, minimum living wages, job programs, and affordable housing options might enable individuals to confidently provide for their families, which would greatly reduce stress and leave room for caregivers to focus on other aspects of life, such as pursuing educational opportunities or spending quality time with their children.

Although there is growing interest in advancing these kinds of federal policies for the general population, many immigrant families are not eligible for certain existing government initiatives (30). For example, households with mixed immigration status families were excluded from federal COVID-19 economic relief efforts such as the CARES act (31), leaving many in precarious financial situations. Studies of local-level unconditional cash transfer efforts to support households that were ineligible for the CARES act found significant improvement in mental health among recipients (32), suggesting that policies that alleviate food and housing insecurity could support mental health in immigrant communities. Other supportive policies specific to immigrant communities, such as legal support to address restrictive immigration policies and enforcement practices, and advocacy for humane immigration policies, may be helpful in reducing stress and promoting mental health as well. However, immigrant communities are heterogeneous, and additional research is needed to better understand which policies might have the greatest mental health impact for different immigrant groups (33), and how some policies may need to be tailored to ensure that immigrant families are not excluded.

## Equitable access to mental health prevention and treatment

While necessary, increasing general support through inclusive federal, state and local policies will not be sufficient to improve the mental health of all immigrant families. Investment in preventive interventions can also help equip families, educators, and healthcare providers with the tools they need to support mental health. To truly promote health equity, preventive interventions should be designed with and for racially and culturally diverse communities (34). When interventions are not co-created in this context, there is a need to evaluate fit, access and effectiveness with the targeted community, and in most cases thoughtful adaptations will be necessary to achieve the desired impact. Although the literature highlights the tension between adhering to fidelity of evidence-based interventions (EBIs) and adapting EBIs to specific stakeholder groups, many scholars have concluded that it is feasible and imperative to do both (35). For EBIs that were not developed for a specific population, the core concepts may seem relevant but framing and implementation often need to be culturally adapted to fully engage the community and ultimately attain optimal outcomes.

Several evidence-based preventive interventions (EBIs) developed specifically for immigrant families, like Madres a Madres (34) and Abriendo Puertas (36), have been shown to be effective. Further, researchers across the country have described cultural adaptations of existing EBIs (37). Similarly, EBIs that are part of TGS, including ParentCorps (38), Reach Out and stay Strong, Essentials for new mothers (ROSE) (39), PlayReadVIP (formerly known as VIP) (40), and Healthy Steps (41), have been either developed with or adapted for families of young children in culturally-relevant ways that promote mental health. For example, ROSE, a postpartum depression prevention intervention, was originally developed and tested with Latinx and Black women. As part of TGS, the intervention has been adapted for the Chinese immigrant community in Sunset Park through deep community engagement, manual adaptations, and iterative rounds of pilot testing. The adaptation process (42, 43) included a literature review in both Chinese and English languages, interviews with mental health experts in the Chinese-American community, community focus groups that informed intervention modifications, and pilots of the culturally adapted intervention. Although the core, evidence-based components remain, the adapted version is framed to meaningfully engage and center the Chinese immigrant community (e.g., “self-care” is reframed to “creating balance and harmony”). Ongoing research is evaluating whether this adapted version leads to desired outcomes with Chinese immigrant families.

Unfortunately, preventive EBIs are often difficult to sustain in immigrant communities due to cost, as many immigrants do not have any insurance coverage (5) and for those who have Medicaid, reimbursing preventive behavioral health services is challenging (44). Many programs are, in part, often supported by private dollars, but this is not sustainable or scalable. Enabling sustainable, billable support for preventive behavioral health programs is critical, and can be efficient and cost-effective, leading to savings in multiple sectors of society in the long-run (45). Expanding Medicaid to include more coverage of preventive behavioral health services would greatly assist in this effort.

The federal government has supported some pregnant and parenting people by funding distinct programs. For example, The Maternal, Infant, and Early Childhood Home Visiting (MIECHV) Program funds home visiting programs among families facing increased risk (46), and Head Start, a long-standing federally-funded early childhood program, mandates evidence-based parenting programs in under-resourced communities (47). At the state level, the California Medicaid Program (MediCal) funds the Comprehensive Perinatal Services Program (CPSP), which provides enhanced services, including psychosocial counseling, to patients (48). Similar funding opportunities should be expanded to include other preventive services and populations. Hospital systems’ community benefit plans could also offer an innovative way to invest in immigrant communities and fund preventive services. While many hospitals primarily conceive of their community benefit efforts as providing financial assistance to patients (49), under the Affordable Care Act (ACA), not-for-profit hospitals are required to assess community need and develop programs to address needs that are identified (50). In Sunset Park, for example, NYU Langone Health’s community benefit plan helps fund some of the TGS programs mentioned above.

Further, communities throughout the U.S. have limited access to mental health services, in large part because of the dearth of mental health professionals (51). This problem is exacerbated in immigrant communities where there is a need for mental health professionals who speak languages other than English and are representative of diverse communities. Because mental health has distinct conceptualizations in different cultures, access to culturally responsive and linguistically aligned services from providers who are skilled in centering families’ values, beliefs, and lived racialized experiences is critical to high-quality and equitable care (52). Funding must be available to hire and retain mental health professionals who reflect the immigrant communities they serve. Investing in linguistically and culturally diverse young people from immigrant communities and encouraging them to pursue careers in mental health is one specific way to address the culturally reflective service provider gap. This includes, but is not limited to, providing opportunities for youth to learn about different career options and boosting financial assistance for internships and academic programs. Federal and state-level scholarship programs could support this approach, and the ACA requirements for Community Benefit Plans could be leveraged to address this gap. Expanding the workforce to include peer-providers and foreign-trained mental health professionals would also enable greater access to culturally aligned providers. Municipal authorities should explore procedures for credentialing and compensating both of these untapped community resources, and they should be included in Medicaid reimbursement plans.

In addition, enhanced cultural training would enable all mental health professionals to understand how different immigrant communities conceptualize mental health—including the meaning of different symptoms and help-seeking behaviors. Cultural values and norms can impact how mental health is understood, and can lead to stigmatization (53, 54). For example, individuals from some immigrant backgrounds hold beliefs that mental health issues not be communicated outside a tight-knit group of family and friends (55); in other cultures, disclosure of mental illness to family and friends is dissuaded (54). Cultural mental health competency and humility can help providers address stigma related to mental health and identify culturally relevant ways to engage communities in mental health prevention and treatment.

Cultural competency, however, is not the only important lens in mental health care. Families face intersecting social vulnerabilities (56), including those related to gender, sexual orientation, legal status, religion and others. Immigrant families may be part of multiple marginalized groups; understanding how these interact in an intersectional framework that recognizes multiple levels of oppression is essential to providing mental health care in immigrant communities.

Academic programs that train mental health service professionals need to ensure that their curricula emphasize the importance of recognizing and addressing structures and power dynamics that shape clinical interactions, such as racism and many other forms of bias (57). It is recognized that mental health providers require specialized training in the mental health disorders most prevalent in the communities they serve. In immigrant communities, this means being prepared to recognize and treat distress, mental health symptoms and disorders related to pre-and post-immigration trauma (4). Accrediting institutions should consider ways to ensure that issues of racism, immigration trauma, and gender and sexual orientation equity are meaningfully included in training curricula.

Even when mental health services are available by providers with cultural competence and humility, barriers to utilizing care exist for many immigrant families. Studies underscore numerous logistical barriers to care, such as cost, transportation, childcare, and hours of operation (58). During the pandemic, telemedicine was successfully offered as a way to address barriers in accessing care (59). However, this mode of service delivery presents additional barriers for families who do not have access to technology, have low digital literacy skills, have limited or no access to broadband, or do not have privacy in their homes (60). Nationally, one in 10 families headed by Hispanic immigrants had no access to the internet in 2016. Similarly, in Sunset Park, Brooklyn, 15% of households do not have internet access, in stark contrast to 5% in a neighboring community with a smaller immigrant popultion (61). Even when families do have access to the internet, connection speed for many is slow (62), rendering meaningful use of websites and apps nearly impossible. While the availability of virtual services should continue post-pandemic to help families access care, relying on this mode as a substitute for in-person care delivery leaves out some of the most under-resourced families (59). Ensuring that mental health services address the broad range of psychological, technical and logistical barriers faced by immigrant families is essential to achieving mental health equity.

## Discussion

Mental health is a critical component of long, healthy and fulfilling lives. Focusing on inclusive policies and tailored programming that create equitable environments where people can provide for themselves and their families might help reduce stress and prevent many mental health problems among immigrant communities. For some, this type of support will not be enough and prevention and treatment services are critical to promote mental wellness. Mental health providers should be linguistically and culturally representative of the community, allowing for maximum comfort, mutual understanding, and equitable access to services. With a multi-pronged approach involving policies and programs to promote mental health and prevent and treat mental health problems and disorders, immigrant communities can get what they need, when they need it, and all families can be supported to thrive.

## Data availability statement

Publicly available datasets were analyzed in this study. We analyzed census data in this article (data.census.gov).

## Ethics statement

The studies involving humans were approved by NYU Langone Institutional Review Board. The studies were conducted in accordance with the local legislation and institutional requirements. The participants provided their written informed consent to participate in this study.

## Author contributions

BK: Conceptualization, Funding acquisition, Writing – original draft, Writing – review & editing. RB-G: Writing – original draft, Writing – review & editing. NR: Writing – original draft, Writing – review & editing. JN: Writing – original draft, Writing – review & editing. LB: Conceptualization, Writing – original draft, Writing – review & editing.

## References

[1] Ward N Batalova J (2023). Frequently requested statistics on immigrants and immigration in the United States. Washington, DC: Migration Policy Institute. Available at: https://www.migrationpolicy.org/article/frequently-requested-statistics-immigrants-and-immigration-united-states#:~:text=How%20many%20U.S.%20residents%20are,or%2020%20percent)20from202010 (Accessed January 19, 2024).

[2] Cardoso JB Thompson SJ. Common themes of resilience among Latino immigrant families: a systematic review of the literature. Fam Soc. (2010) 91:257–65. doi: 10.1606/1044-3894.4003

[3] Rios Casas F Ryan D Perez G Maurer S Tran AN Rao D. “Se vale llorar y se vale reír”: Latina immigrants’ coping strategies for maintaining mental health in the face of immigration-related stressors. J Racial Ethn Health Disparities. (2020) 7:937–48. doi: 10.1007/s40615-020-00717-7, PMID: 32040841 PMC7416525

[4] Sangalang CC Becerra D Mitchell FM Lechuga-Peña S Lopez K Kim I. Trauma, post-migration stress, and mental health: a comparative analysis of refugees and immigrants in the United States. J Immigr Minor Health. (2019) 21:909–19. doi: 10.1007/s10903-018-0826-2, PMID: 30244330

[5] Capps R Fix M Ost J. (2004). The health and well-being of young children of immigrants. Washington, DC: Urban Institute. Available at: https://www.aber.ac.uk/en/media/departmental/sell/pdf/wellbeinghealth/THE-HEALTH-AND-WELL-BEING-OF-YOUNG-CHILDREN-OF-IMMIGRANTS.pdf (Accessed November 1, 2023).

[6] Bernstein H Gonzalez D Karpman M. (2020). Amid confusion over the public charge rule, immigrant families continued avoiding public benefits in 2019. Washington, DC: Urban Institute. Available at: https://www.urban.org/sites/default/files/publication/102221/amid-confusion-over-the-public-charge-rule-immigrant-families-continued-avoiding-public-benefits-in-2019_3.pdf (Accessed August 16, 2022).

[7] Manning C Gregoire A. Effects of parental mental illness on children. Psychiatry. (2006) 5:10–2. doi: 10.1383/psyt.2006.5.1.10

[8] Ormiston CK Chiangong J Williams F. The COVID-19 pandemic and Hispanic/Latina/o immigrant mental health: why more needs to be done. Health Equity. (2023) 7:3–8. doi: 10.1089/heq.2022.0041 36744233 PMC9892920

[9] Thomeer MB Moody MD Yahirun J. Racial and ethnic disparities in mental health and mental health care during the COVID-19 pandemic. J Racial Ethn Health Disparities. (2023) 10:961–76. doi: 10.1007/s40615-022-01284-9 35318615 PMC8939391

[10] Castillo EG Ijadi-Maghsoodi R Shadravan S Moore E Mensah MO 3rd Docherty M. Community interventions to promote mental health and social equity. Focus. (2020) 18:60–70. doi: 10.1176/appi.focus.18102, PMID: 32015729 PMC6996071

[11] Vitiello D. (2022). As red states send migrants to blue states, sanctuary cities are crucial. Washington Post. Available at: https://www.washingtonpost.com/made-by-history/2022/09/15/red-states-send-migrants-blue-states-sanctuary-cities-are-crucial/ (Accessed March 25, 2024).

[12] Hogan G Holliday R. What exactly is a Sanctuary City and what does that mean for NYC? The City; February 13, 2024. Available at: https://www.thecity.nyc/2024/02/13/sanctuary-city-explainer-nyc/ (Accessed March 26, 2024).

[13] Hatzenbuehler ML Prins SJ Flake M Philbin M Frazer MS Hagen D. Immigration policies and mental health morbidity among Latinos: a state-level analysis. Soc Sci Med. (2017) 174:169–78. doi: 10.1016/j.socscimed.2016.11.040, PMID: 28043019 PMC5258771

[14] US Census Bureau, American Community Survey 2022 1-year estimates (2024). Table S0501 selected characteristics of the native and foreign-born population. Available at: https://data.census.gov/table/ACSST1Y2022.S0501?q=Native%20and%20Foreign%20Born%20language (Accessed March 27, 2024).

[15] United States Census Bureau (2017). American community survey, 2017-2021. New York City. Available at: https://popfactfinder.planning.nyc.gov/ (Accessed January 16, 2024).

[16] Kerker BD Rojas NM Dawson-McClure S Gonzalez C. Re-imagining early childhood education and school readiness for children and families of color in the time of COVID-19 and beyond. Am J Health Promot. (2023) 37:270–3. doi: 10.1177/08901171221140641c, PMID: 36646660

[17] Barajas-Gonzalez RG Linares Torres H Urcuyo A Salamanca E Kourousias L. Racialization, discrimination, and depression: a mixed-method study of the impact of an anti-immigrant climate on Latina immigrant mothers and their children. Health. (2022) 2:100084. doi: 10.1016/j.ssmmh.2022.100084

[18] Gover AR Harper SB Langton L. Anti-Asian hate crime during the COVID-19 pandemic: exploring the reproduction of inequality. Am J Crim Justice. (2020) 45:647–67. doi: 10.1007/s12103-020-09545-1 32837171 PMC7364747

[19] Duric V Clayton S Leong ML Yuan LL. Comorbidity factors and brain mechanisms linking chronic stress and systemic illness. Neural Plast. (2016) 2016:1–16. doi: 10.1155/2016/5460732, PMID: 26977323 PMC4761674

[20] Hernandez A Plant EA Sachs-Ericsson N Joiner TE Jr. Mental health among Hispanics and Caucasians: risk and protective factors contributing to prevalence rates of psychiatric disorders. J Anxiety Disord. (2005) 19:844–60. doi: 10.1016/j.janxdis.2004.11.002, PMID: 16243634

[21] Frankham C Richardson T Maguire N. Psychological factors associated with financial hardship and mental health: a systematic review. Clin Psychol Rev. (2020) 77:101832. doi: 10.1016/j.cpr.2020.101832 32088498

[22] Vargas ED Sanchez GR Juárez M. Fear by association: perceptions of anti-immigrant policy and health outcomes. J Health Polit Policy Law. (2017) 42:459–83. doi: 10.1215/03616878-3802940 28213396 PMC6624117

[23] Rothrock NE Amtmann D Cook KF. Development and validation of an interpretive guide for PROMIS scores. J Patient Rep Outcomes. (2020) 4:1–7. doi: 10.1186/s41687-020-0181-7 PMC7048882 32112189

[24] Allen J Balfour R Bell R Marmot M. Social determinants of mental health. Int Rev Psychiatry. (2014) 26:392–407. doi: 10.3109/09540261.2014.928270 25137105

[25] White LK Kornfield SL Himes MM Forkpa M Waller R Njoroge WFM. The impact of postpartum social support on postpartum mental health outcomes during the COVID-19 pandemic. Arch Womens Ment Health. (2023) 26:531–41. doi: 10.1007/s00737-023-01330-3, PMID: 37268777 PMC10238239

[26] Coburn SS Gonzales NA Luecken LJ Crnic KA. Multiple domains of stress predict postpartum depressive symptoms in low-income Mexican American women: the moderating effect of social support. Arch Womens Ment Health. (2016) 19:1009–18. doi: 10.1007/s00737-016-0649-x, PMID: 27329119 PMC5106307

[27] Corona K Yang T Dunton G Toledo-Corral C Grubbs B Eckel SP. The role of social support and acculturation factors on postpartum mental health among Latinas in the MADRES pregnancy cohort. J Immigr Minor Health. (2024) 26:72–80. doi: 10.1007/s10903-023-01542-w, PMID: 37897652 PMC10771371

[28] Misra S Kwon SC Abraído-Lanza AF Chebli P Trinh-Shevrin C Yi SS. Structural racism and immigrant health in the United States. Health Educ Behav. (2021) 48:332–41. doi: 10.1177/10901981211010676, PMID: 34080482 PMC8935952

[29] Doran EL Bartel AP Ruhm CJ Waldfogel J. California's paid family leave law improves maternal psychological health. Soc Sci Med. (2020) 256:113003. doi: 10.1016/j.socscimed.2020.113003, PMID: 32464413

[30] Acevedo-Garcia D Joshi P Ruskin E Walters AN Sofer N. Restoring an inclusionary safety net for children in immigrant families: a review of three social policies. Health Aff. (2021) 40:1099–107. doi: 10.1377/hlthaff.2021.00206, PMID: 34228532

[31] Migration Policy Institute (2020). Mixed-status families ineligible for CARES act federal pandemic stimulus checks. Available at: https://www.migrationpolicy.org/content/mixed-status-families-ineligible-pandemic-stimulus-checks (Accessed July 7, 2022)

[32] Kumar SL Calvo-Friedman A Freeman AL Fazio D Johnson AK Seiferth F. An unconditional cash transfer program for low-income new Yorkers affected by COVID-19. J Urban Health. (2023) 100:16–28. doi: 10.1007/s11524-022-00693-9, PMID: 36224486 PMC9555690

[33] Nieri T Ramachandran M Bruckner T Link B Ayón C. Sanctuary city policies and Latinx immigrant mental health in California. SSM Popul Health. (2023) 21:101319. doi: 10.1016/j.ssmph.2022.101319, PMID: 36589276 PMC9798158

[34] Williamson AA Knox L Guerra NG Williams KR. A pilot randomized trial of community-based parent training for immigrant Latina mothers. Am J Community Psychol. (2014) 53:47–59. doi: 10.1007/s10464-013-9612-4, PMID: 24276907

[35] Castro FB Barrera M Martinez CR. The cultural adaptation of prevention interventions: resolving tensions between fidelity and fit. Prev Sci. (2004) 5:41–5. doi: 10.1177/1049731514535989 15058911

[36] Caal S Moore K Murphy K Lawner E Rojas A DeMand A. Abriendo Puertas: evaluation of a parent education program for Latinos. Crit Sociol. (2019) 41. doi: 10.1177/08969205231188737

[37] Le HN Zmuda J Perry DF Muñoz RF. Transforming an evidence-based intervention to prevent perinatal depression for low-income Latina immigrants. Am J Orthop. (2010) 80:34. doi: 10.1111/j.1939-0025.2010.01005.x 20397987

[38] Brotman LM Dawson-McClure S Kamboukos D Huang KY Calzada EJ Goldfeld K. Effects of ParentCorps in prekindergarten on child mental health and academic performance: follow-up of a randomized clinical trial through 8 years of age. JAMA Pediatr. (2016) 170:1149–55. doi: 10.1001/jamapediatrics.2016.1891, PMID: 27695851 PMC5642293

[39] Zlotnick C Tzilos G Miller I Seifer R Stout R. Randomized controlled trial to prevent postpartum depression in mothers on public assistance. J Affect Disord. (2016) 189:263–8. doi: 10.1016/j.jad.2015.09.059, PMID: 26454186 PMC4641029

[40] Cates CB Weisleder A Dreyer BP Berkule Johnson S Vlahovicova K Ledesma J. Leveraging healthcare to promote responsive parenting: impacts of the video interaction project on parenting stress. J Child Fam Stud. (2016) 25:827–35. doi: 10.1007/s10826-015-0267-7, PMID: 27134514 PMC4847426

[41] Johnston BD Huebner CE Tyll LT Barlow WE Thompson RS. Expanding developmental and behavioral services for newborns in primary care: effects on parental well-being, practice, and satisfaction. Am J Prev Med. (2004) 26:356–66. doi: 10.1016/j.amepre.2003.12.018, PMID: 15110063

[42] Lee M Kwon SC. Participatory dissemination and implementation research in community settings In: Kwon SC Trinh-Shevrin C Islam NS Yi SS, editors. Applied Population Health Approaches for Asian American Communities. Hoboken, NJ: Jossey Bass (2022). 121–32.

[43] Min D. Case 1. REACH FAR: cultural adapted, evidence-based hypertension control programs among Asian Americans in New York and New Jersey In: Kwon SC Trinh-Shevrin C Islam NS Yi SS, editors. Applied Population Health Approaches for Asian American Communities. Hoboken, NJ: Jossey Bass (2022). 231–6.

[44] Weist MD Goldstein J Evans SW Lever NA Axelrod J Schreters R. Funding a full continuum of mental health promotion and intervention programs in the schools. J Adolesc Health. (2003) 32:70–8. doi: 10.1016/S1054-139X(03)00067-3 12782445

[45] Arango C Díaz-Caneja CM McGorry PD Rapoport J Sommer IE Vorstman JA. Preventive strategies for mental health. Lancet Psychiatry. (2018) 5:591–604. doi: 10.1016/S2215-0366(18)30057-9 29773478

[46] HRSA Maternal and Child Health. Maternal, infant, and early childhood home visiting (MIECHV) program. Available at: https://mchb.hrsa.gov/programs-impact/programs/home-visiting/maternal-infant-early-childhood-home-visiting-miechv-program (Accessed March 25, 2024).

[47] Head Start Early Childhood Learning and Knowledge Center (2024). Head start policy and regulations. Available at: https://eclkc.ohs.acf.hhs.gov/policy/head-start-act (Accessed March 25, 2024).

[48] Lopes SS Shi A Li J. California’s comprehensive perinatal services program and birth outcomes. Front Public Health. (2023) 11:1321313. doi: 10.3389/fpubh.2023.1321313 38179565 PMC10764413

[49] James J. (2016). Nonprofit hospitals' community benefit requirements. Health affairs. Washington, DC, USA: project HOPE; February 25, 2016. Available at: https://www.healthaffairs.org/do/10.1377/hpb20160225.954803/ (Accessed March 25, 2024).

[50] The Patient Protection and Affordable Care Act (PPACA) (2010). Pub. L. No. 111–148, 124 stat. 119. United States government. Community health needs assessments. US government publishing office (2015). 26 CFR sec 1.501(r)-3.

[51] KFF (2023). Mental health care health professional shortage. Available at: Mental Health Care Health Professional Shortage Areas (HPSAs) | KFF (Accessed November 21, 2023).

[52] Sanchez K Ybarra R Chapa T Martinez ON. Eliminating behavioral health disparities and improving outcomes for racial and ethnic minority populations. Psychiatr Serv. (2016) 67:13–5. doi: 10.1176/appi.ps.201400581, PMID: 26325461

[53] Derr AS. Mental health service use among immigrants in the United States: a systematic review. Psychiatr Serv. (2016) 67:265–70. doi: 10.1176/appi.ps.201500004 26695493 PMC5122453

[54] Eghaneyan BH Murphy ER. Measuring mental illness stigma among Hispanics: a systematic review. Stigma Health. (2020) 5:351–63. doi: 10.1037/sah0000207

[55] Li JY. Acculturation and social stigma: mental health communicative action and help-seeking behaviors among Chinese immigrants in the United States. Int J Strateg Commun. (2021) 15:487–503. doi: 10.1080/1553118X.2021.1984918

[56] Viruell-Fuentes EA Miranda PY Abdulrahim S. More than culture: structural racism, intersectionality theory, and immigrant health. Soc Sci Med. (2012) 75:2099–106. doi: 10.1016/j.socscimed.2011.12.037 22386617

[57] Metzl JM Hansen H. Structural competency: theorizing a new medical engagement with stigma and inequality. Soc Sci Med. (2014) 103:126–33. doi: 10.1016/j.socscimed.2013.06.032 24507917 PMC4269606

[58] Alvidrez J Azocar F. Distressed women’s clinic patients: preferences for mental health treatments and perceived obstacles. Gen Hosp Psychiatry. (1999) 21:340–7. doi: 10.1016/s0163-8343(99)00038-9 10572775

[59] Costa M Reis G Pavlo A Bellamy C Ponte K Davidson L. Tele-mental health utilization among people with mental illness to access care during the COVID-19 pandemic. Community Ment Health J. (2021) 57:720–6. doi: 10.1007/s10597-021-00789-7, PMID: 33566269 PMC7873669

[60] Sevelius JM Gutierrez-Mock L Zamudio-Haas S McCree B Ngo A Jackson A. Research with marginalized communities: challenges to continuity during the COVID-19 pandemic. AIDS Behav. (2020) 24:2009–12. doi: 10.1007/s10461-020-02920-3, PMID: 32415617 PMC7228861

[61] United States Census Bureau (2017). American community survey 5-year estimates, table B28002, 2017-2021. New York City. Available at: https://data.census.gov/ (Accessed May 1, 2023).

[62] Katz VS Gonzalez C Clark K. Digital inequality and developmental trajectories of low-income, immigrant, and minority children. Pediatrics. (2017) 140:S132–S136.z. doi: 10.1542/peds.2016-1758R 29093048

